# Reasons for Delayed Carotid Endarterectomy for Symptomatic Carotid Stenosis in Norway 2018–2019: A National Audit

**DOI:** 10.1016/j.ejvsvf.2025.07.003

**Published:** 2025-07-08

**Authors:** Martin Altreuther, Celine Harlinn Sørlie, Benedicte Skaug Hansen, Christian Lyng, Karsten Myhre, Toril Rabben, Ramez Bahar, Tonje Berglund, Dorte Bundgaard, Erik Mulder Pettersen

**Affiliations:** a Department of Vascular Surgery, St Olavs Hospital, Trondheim, Norway; b Norwegian Registry for Vascular Surgery (NORKAR), Trondheim, Norway; c Institute for Circulation and Medical Imaging, NTNU, Trondheim, Norway; d Department of Vascular Surgery, Haukeland University Hospital, Bergen, Norway; e Department of Vascular Surgery, Vestfold Hospital, Tønsberg, Norway; f Department of Vascular Surgery, Innlandet Hospital, Hamar, Norway; g Department of Vascular Surgery, Vestre Viken Hospital, Drammen, Norway; h Department of Vascular Surgery, Oslo University Hospital Aker, Oslo, Norway; i Department of Vascular Surgery, Tromsø University Hospital, Tromsø, Norway; j Department of Vascular Surgery, Akershus University Hospital, Akershus, Norway; k Department of Vascular Surgery, Østfold Hospital, Kalnes, Norway; l Department of Surgery, Sørlandet Hospital, Kristiansand, Norway

**Keywords:** Carotid endarterectomy, Symptomatic carotid stenosis, Delayed CEA for symptomatic stenosis, Vascular registry research

## Abstract

**Objective:**

Symptomatic carotid stenosis is one of the main causes of *amaurosis fugax*, transient ischaemic attack (TIA), and stroke. National and international guidelines recommend treatment with carotid endarterectomy (CEA) within 14 days of the index event. In Norway, the proportion of patients operated on within 14 days increased from 65% in 2015 to 83% in 2020. A national clinical audit cross sectional study was performed to identify the reasons for delayed CEA that could be addressed by quality improvement.

**Methods:**

Patients operated on by CEA for symptomatic stenosis more than 14 days after the index event in 2018 and 2019 were identified from the Norwegian Registry for Vascular Surgery. The local registrar assessed the reason for the delay, based on the medical record. Possible reasons for delay were categorised as medical reasons, doctor delay, patient delay, and other reasons.

**Results:**

Fourteen units performed 686 CEA for symptomatic stenosis in Norway in the study period, of which 179 (26%) were delayed. Ten units participated in the audit, accounting for 120 of 179 (67%) delayed CEAs. The reason for delay was identified for all patients in the participating units. There was a medical reason for the delay in 23 patients. There was doctor delay in 54 cases, patient delay in 28 cases, and a combination of patient delay and doctor delay in 10 cases. The reason for the delay was travel abroad in five cases.

**Conclusion:**

Delayed CEA for symptomatic stenosis is usually due to doctor delay or patient delay. Medical reasons account for 19% of delayed operations. This implies that quality improvement is feasible by addressing doctor and patient delay. Healthcare providers should implement strategies to decrease the proportion of delayed CEA for symptomatic stenosis. Patient delay should be addressed with regular information campaigns.

## Highlights

• A national audit of the reasons for delayed carotid endarterectomy (CEA) in Norway for 2018 and 2019 was performed.
• Delayed CEA was due to medical reasons in slightly more than 19% of all cases.
• The main reasons for delayed CEA were doctor (45%) and patient delays (23%).
• Quality improvement should aim to reduce doctor and patient delays.

## INTRODUCTION

Carotid stenosis can be the cause of *amaurosis fugax*, transient ischaemic attack (TIA), and stroke, usually due to thromboembolic events. Carotid endarterectomy (CEA) reduces the risk of new TIA or stroke in these patients. National and international guidelines recommend that symptomatic carotid stenosis is treated with CEA within 14 days of the index event.^1^, ^2^, ^3^, ^4^ There are recommendations not to operate within 48 hours,^5^^,^^6^ and one publication recommends stratification by a modified Rankin scale score for optimal timing.^7^

Not all patients can be operated on within the recommended period, due to medical conditions and contraindications like cardiac comorbidity, or a large area of infarction conferring increased risk of intracranial bleeding in the peri-operative period. In these cases, delayed operation is recommended, although there is no definitive evidence for how long this delay should be.^1^^,^^2^ The proportion of patients with symptomatic carotid stenosis operated on within 14 days after the index event is a national quality indicator for vascular surgery in Norway.^8^ The benchmark for high quality is defined as 80% of patients with symptomatic carotid stenosis treated within 14 days of the index event;^9^ however, the variation in this quality indicator between the different vascular units is large.^9^

Treatment for TIA and stroke in Norway is organised through the Norwegian National Care Pathway for Stroke Treatment. Acute patients are typically admitted to stroke units or referred to TIA clinics in case of minor transient symptoms. Virtually all local and central hospitals have stroke units, 50 in total; 95% of the stroke patients in Norway in 2020 were treated at a stroke unit.^10^ Symptomatic carotid stenosis is typically diagnosed in the workup of patients with TIA or stroke at the stroke units, including Duplex ultrasound, CTA of the cervical vessels, and cerebral magnetic resonance imaging (MRI); patients with symptomatic carotid stenosis are referred to vascular surgery for assessment for CEA. The cooperation between the stroke and vascular units is well established and part of the National Care Pathway for Stroke Treatment.

**Figure 1:**
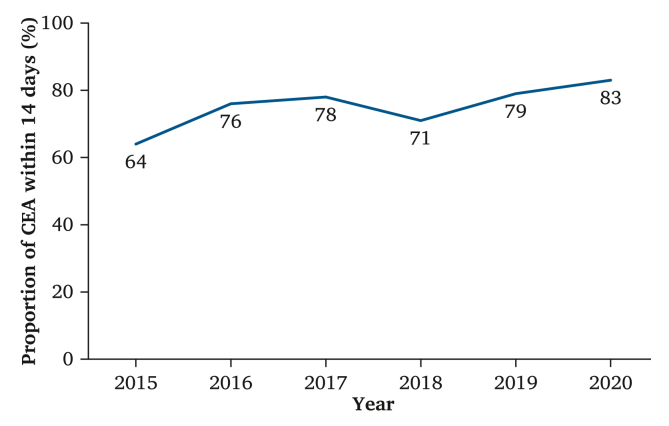
The proportion of carotid endarterectomy (CEA) for symptomatic stenosis performed within 14 days of the index event 2015–2020. National Quality Indicator for Vascular Surgery in Norway.

On a national level, the proportion of patients with symptomatic carotid stenosis treated within 14 days has increased from 65% in 2015 to 83% in 2020^9^ (Fig. 1). To investigate the reasons for delayed treatment of symptomatic carotid stenosis, a national audit of the reasons for delayed CEA was undertaken, based on the data from the Norwegian Registry for Vascular Surgery for the years 2018 and 2019. The aim of the audit was to identify reasons for delayed operation in patients with symptomatic carotid stenosis that could be addressed with quality improvement, to increase the proportion of patients operated on within 14 days of the index event.

## METHODS

Patients with symptomatic carotid stenosis who were operated on more than 14 days after the index event in 2018 and 2019 were identified from the Norwegian Registry for Vascular Surgery. The local registry representative was given a list with the globally unique identifier (GUID) codes for the actual registrations for identification in the local registry. The reasons for delay were identified from the electronic patient record, and categorised as medical reason, doctor delay, patient delay, or other reason, with a free text description.

Medical reason was defined as a medical contraindication for CEA within 14 days, such as a large or disabling stroke, according to recommendation 46 in the current carotid guidelines,^2^ or comorbidity indicating further evaluation and treatment before carotid surgery. Doctor delay was defined as delayed medical referral or long waiting time for specialist assessment or operation that made timely treatment impossible. Patient delay was defined as delayed contact with the healthcare system that made timely treatment impossible. The local registrar performed the categorisation based on information in the electronic patient record.

This study also performed a subgroup analysis for patients with stroke or TIA as an indication for CEA, to investigate whether there were differences in the proportion of delayed operations between the subgroups. Differences between the subgroups were assessed with chi squared test.

### Ethical considerations

The Norwegian Registry for Vascular Surgery is a part of the Norwegian Cardiovascular Disease Registry, with legal permission to continuously analyse registry data. The current study is an outcome analysis only, with the aim of quality improvement. Assessment and approval by the regional ethical committee was therefore unnecessary.

## RESULTS

Fourteen units performed 686 CEA for symptomatic stenosis in Norway in the study period, of which 507 (75%) were within 14 days and 179 (26%) were delayed. The 179 operations with delayed CEA for symptomatic stenosis were identified from the registry. Of the 14 vascular units that perform CEA in Norway, 10 participated in the audit, and the reason for delayed CEA for symptomatic stenosis was identified in all 120 cases operated on at the participating units in the study period, while the 59 cases at the four remaining units were not included. The participating units are listed in Table 1.

**Table 1:** Participating units and number of patients included (*n* = 120).

Unit	Patients
Akershus University Hospital	<5
Oslo University Hospital, Aker	6
Haukeland University Hospital	33
Drammen Hospital	9
Innlandet Hospital, Hamar	10
Østfold Hospital, Kalnes	<5
Sørlandet Hospital, Kristiansand	<5
St. Olavs Hospital	19
Tromsø University Hospital	<5
Vestfold Hospital, Tønsberg	29
Total	120

Data are shown as *n*.

There was a medical reason for the delay in 23 (19.2%) patients, doctor delay in 54 (45%) patients, patient delay in 28 (23.3%) patients, and a combination of both in 10 (8.3%) patients. The reason for the delay in five (4.2%) cases was that the patients were on holiday abroad at the time of the index event, delaying pre-operative assessment and surgery until they had returned home. Table 2 shows an overview of the results.

**Table 2:** Reasons for delayed carotid endarterectomy (CEA).

Reason	Patients
Medical	23 (19.2)
Doctor delay	54 (45)
Patient delay	28 (23.3)
Both doctor and patient delay	10 (8.3)
Other (travel abroad)	5 (4.2)

Data are shown as *n* (%).

The medical reason for the delay was the need for further interdisciplinary assessment in seven patients, cardiac disease indicating further diagnostic or therapeutic procedures in five patients, while major stroke, infection, or other reasons caused delay in fewer than five patients each.

Doctor delay was due to waiting time for assessment at the vascular outpatient clinic or for the operation in 18 patients, delayed referral from general practitioner (GP) to ophthalmologist or neurologist in 15 patients, and delayed referral for operation after specialist assessment in 19 patients. In some patients, several specialities were involved in the delay. Table 3 shows an overview of the results.

**Table 3:** Specialty responsible for doctor delay (main responsibility).

Specialty		Description
Vascular surgery	18 (33.3)	Waiting time for assessment or surgery
General practitioner	15 (27.8)	Delayed referral to neurologist or ophthalmologist
Neurology	9 (16.7)	Waiting time or delayed referral. Some patients not referred to surgery
Ophthalmology	5 (9.3)	Waiting time or delayed referral
Internal medicine	5 (9.3)	Delayed referral, waiting time for MRI
Uncertain	<5 (<9.3)	Uncertain main responsibility

Data are shown as *n* (%). MRI = magnetic resonance imaging.

Patient delay was usually due to late contact with the GP, in 22 patients in total, while six patients wanted a delayed operation for personal reasons, such as planned family events. Patient delay was the most common reason for delayed CEA for symptomatic carotid stenosis at the vascular units in Hamar (eight patients, 80%) and Trondheim (nine patients, 47%), but less common at all other hospitals (11 patients in eight hospitals, 0–33%).

The subgroup analysis for the patients with TIA and stroke showed that 57 of 275 patients (20.7%) with the indication stroke had a delayed CEA. For patients with the indication TIA, the proportion with delayed operation was 122 of 411 patients (29.7%). The difference was statistically significant, with a *p* value of <0.008.

## DISCUSSION

This national audit investigated the reasons for delayed CEA in Norway in the years 2018 and 2019 and found that 179 of 686 (26%) CEAs for symptomatic stenosis were delayed. The reason for delay could be identified for all 120 patients at the 10 participating units. The most common reasons for delay were doctor delay in 54 (45%) patients and patient delay in 28 (23.3%) patients, or a combination of both in 10 (8.3%) patients. In 23 (19.2%) patients, there was a medical reason for the delay and in five (4.2%) cases, the reason for the delay was holiday travel abroad at the time of the index event.

Subgroup analysis showed that the proportion of delayed CEA was statistically significantly higher for patients with the TIA indication compared with the stroke indication (29.7% *vs*. 20.7%; *p* < 0.01). This finding may be due to the transient symptoms of TIA and could be an argument for a new public information campaign, stressing that it is important to seek medical help even if the symptoms for stroke are transient. However, this audit did not address this aspect because it focused on the reasons for delay for the whole group.

Timely treatment of symptomatic carotid stenosis is an important quality indicator, and the introduction of the term ‘time is brain’ has indeed increased attention on timely treatment for symptomatic carotid stenosis.^11^ This has been addressed in the European Society for Vascular Surgery guidelines,^1^^,^^2^ as well as with information campaigns in several countries, such as the ‘FAST’ campaign in the UK^12^ or the ‘Talk-Smile-Lift’ campaign in Norway.^13^

New national guidelines in Norway^3^ and standards for inpatient care for stroke patients were published in 2017.^4^ Patient information about symptoms and signs for stroke are available online.^13^ This has probably contributed much to the increase in patients with CEA for symptomatic stenosis operated on within 14 days from the index event from about 65% in 2015 to 83% in 2020,^6^ as shown Fig. 1. Compared with a prospective national study in Norway from 2014 – 2015,^14^ where 61.7% of patients with symptomatic carotid stenosis had CEA within 14 days after the index event, the results are greatly improved.

Compared with other countries, the results in Norway are relatively good. The proportion of patients with symptomatic carotid stenosis operated on within 14 days from the index event was 82.9% in Sweden in 2019,^15^ while the 75% percentile in Denmark was 18 days in 2018,^16^ and median time to treatment in the UK was 12 days in 2019.^17^ These data are unavailable in other countries and comparison is not possible.

The results of the national audit also indicate that further quality improvement is possible, mainly because slightly more than 19% of all delayed operations are due to medical reasons where the delay is obviously necessary. The reasons for delay in other countries are unknown, but it is likely that the figures for medical reasons for delayed CEA are similar to the results of this study. There may be different figures for doctor and patient delay in other countries, but both can be addressed with quality improvement. Waiting time for outpatient assessment and late referral can be addressed with improved patient logistics and a care pathway for patients with symptomatic carotid stenosis. Patient delay can be addressed with a new information campaign, similar to the ‘FAST’ campaign in the UK^12^ or the ‘Talk-Smile-Lift’ campaign in Norway.^13^ Waiting time for operation may also be reduced by improved or prioritised capacities for carotid surgery.

The audit did not provide a good explanation for the large variation between units. The observation that two units had a very high proportion of patient delay may indicate a local need for a new public information campaign. However, even though the large variation between units cannot be explained, the indicator clearly shows where focus should be more on timely CEA for symptomatic stenosis.

This study did not investigate which specialties performed the pre-operative assessment of all patents operated within 14 days of the index event. Therefore, it is not possible to draw conclusions whether timely assessment and treatment varies between different specialties. However, delayed treatment with CEA for symptomatic stenosis was rare for patients treated in stroke units (internal medicine), while extra waiting time appears to be associated with assessment in other specialties. This may sometimes be due to non-specific symptoms causing delay in the diagnosis of stroke or symptomatic stenosis. High clinical awareness and a low threshold for further diagnostic procedures like MRI and cervical CTA or ultrasound may reduce the proportion of patients with delayed CEA, but this could also increase the number of negative finding MRIs. Introducing better tools for pre-hospital screening and diagnosis may be useful to reduce unnecessary imaging.^18^

Stroke units have a short waiting time and established logistics allowing for diagnosis and treatment of symptomatic carotid stenosis within the recommended time. They have established routines for the early involvement of vascular surgeons, in accordance with the National Care Pathway for Stroke Treatment.^4^ Therefore, it will be beneficial that most patients with TIA and stroke due to carotid stenosis are referred to and treated at stroke units.

### Conclusions

Focus on timely CEA for symptomatic stenosis and a national quality indicator have led to positive change, with 83% of CEA performed within 14 days of the index event in 2020.

This national audit has shown that delayed CEA for symptomatic stenosis had a medical reason in about 19% of the cases. The most common reason for delayed CEA is doctor delay, at 45%. Patient delay is the second most common reason at 23%.

The key elements to reduce doctor delay are urgent referral to a stroke unit with sufficient capacity for rapid assessment and a vascular unit with sufficient operating room capacity for urgent CEA. Patient delay should be addressed with national information campaigns.

Auditing the reasons for delayed CEA at a unit level may uncover specific reasons that can be addressed locally. Healthcare providers must continuously monitor their results to ensure that as many patients as possible are timeously operated on with CEA for symptomatic stenosis.

## FUNDING

None.

## CONFLICT OF INTEREST

None.
